# Linking prosthodontics and periodontics: An implant success rate

**DOI:** 10.6026/973206300210447

**Published:** 2025-03-31

**Authors:** Manu Sharma, Swati Solanki, Rahul Anand Razdan, Ankita Bhargava, Arpita Srivastava, Ipseeta Menon

**Affiliations:** 1 Department of Dental ,LLRM Govt medical college, Meerut, Uttar Pradesh, India; 2 Department of Prosthodontics, Crown and Bridge, Govt. College of Dentistry, Indore, Madhya Pradesh, India; 3 Department of prosthodontics Crown and Bridge, Index Institute of dental sciences, Indore, Madhya Pradesh, India; 4 Department of public health and dentistry, Government College of dentistry, Indore, Madhya Pradesh, India; 5 Department of Oral Medicine and Radiology, Government college of dentistry, Indore, Madhya Pradesh, India; 6 Department of Public Health Dentistry, Kalinga Institute of Dental Sciences, Bhubaneswar, Odisha, India

**Keywords:** Dental implants, prosthodontics, periodontics, implant stability, interdisciplinary approach

## Abstract

Implant success is significantly influenced by the interplay of prosthodontic and periodontal factors, as demonstrated in this study of 20 subjects undergoing dental implant placement. Key findings highlight a strong correlation between implant stability and periodontal health, particularly gingival health and bone density, while prosthodontic elements such as occlusal load and abutment stability also played pivotal roles. Patients with balanced occlusal forces, stable abutments and healthy periodontal profiles achieved over 90% success in stability and functionality. These results emphasize the importance of an interdisciplinary approach, integrating meticulous prosthodontic planning with robust periodontal maintenance to optimize implant outcomes.

## Background:

Dental implants have become a preferred solution for replacing missing teeth due to their high success rates, longevity and natural functionality. Advancements in implant technology have further solidified their role as a reliable treatment option [1, 2 and 3]. However, successful outcomes require precise clinical planning and the integration of prosthodontic principles for optimal function and aesthetics, alongside periodontal considerations to maintain peri-implant tissue health and stability [3, 4]. From a prosthodontic perspective, implant stability is shaped by factors such as prosthesis type (*e.g.*, single crown, bridge and over denture), occlusal load distribution, material choice and abutment stability [4]. These elements are vital for ensuring the implant withstands functional forces over time. For example, material choice impacts prosthesis durability, while occlusal load distribution influences mechanical stresses on the implant [5]. Abutment stability is particularly critical, as unstable abutments can elevate stress on both the implant and surrounding tissues, potentially resulting in complications like implant loosening or failure [6]. In parallel, periodontal health is paramount for the success and longevity of dental implants. Healthy gingival tissues and adequate bone density are essential for supporting the implant and preventing peri-implant disease, including conditions like peri-implantitis that can compromise implant success [7, 8]. Therefore, it is of interest to show the critical interplay between prosthodontic and periodontal factors in achieving dental implant success.

## Methodology:

This cross-sectional observational study evaluated the impact of prosthodontic and periodontal factors on dental implant success in a sample of 20 patients with functional implants for over six months. Inclusion criteria excluded systemic illnesses affecting implant health and non-compliance with maintenance protocols. Data collection included prosthodontic variables (prosthesis type, occlusal load, material and abutment stability) and periodontal variables (gingival health, pocket depth, bone density). Outcomes measured implant stability, functionality and patient satisfaction. Descriptive statistics, correlation and multiple regression analyses identified key predictors, with a heat map visualizing factor impacts. Ethical approval and informed consent were obtained, but the small sample size and cross-sectional design limit generalizability, warranting larger longitudinal studies for further validation.

## Results and observation:

## Participant demographics:

The study involved 20 participants (12 males and 8 females) with an average age of 45.6 years (range: 35-60 years). All participants had at least one functional dental implant for over six months, with no systemic conditions affecting implant health.

## Descriptive statistics (Table 1):

## Prosthodontic variables:

[1 Type of prosthesis: Single crowns were the most common prosthesis (50%), followed by bridges (30%) and overdentures (20%).

[2] Occlusal load distribution: 60% of participants had moderate occlusal load, while 30% had heavy load and 10% had light load.

[3] Prosthetic material: 40% of prostheses were ceramic, 35% metal-ceramic and 25% zirconia.

[4] Abutment stability: 85% of abutments were stable, while 15% exhibited minor instability.

## Periodontal variables:

[1] Gingival health status: 50% had healthy gingiva, 30% had mild periodontitis and 20% had severe periodontitis.

[2] Periodontal pocket depth: The average pocket depth was 2.4 mm for healthy cases, 3.8 mm for mild periodontitis and 5.6 mm for severe cases.

[3] Bone density at implant site: 55% of participants had medium bone density, 30% had high bone density and 15% had low bone density.

## Correlation analysis:

The Pearson correlation analysis revealed significant associations between periodontal and prosthodontic factors and implant success rates:

[1] Gingival health and implant stability: There was a strong positive correlation (r = 0.78, p < 0.01) between healthy gingiva and implant stability.

[2] Occlusal load and abutment stability: Moderate occlusal loads were positively correlated with abutment stability (r = 0.66, p < 0.05).

[3] Bone density and implant success: Higher bone density was associated with improved implant stability and lower risk of complications (r = 0.72, p < 0.01).

## Regression analysis:

A multiple regression analysis identified the most significant predictors of implant success:

[1] Gingival health (β = 0.48, p < 0.01) and Bone density (β = 0.41, p < 0.05) emerged as the strongest predictors, suggesting that improved gingival health and higher bone density are key factors in implant success.

[2] Occlusal Load had a moderate predictive value (β = 0.35, p < 0.05), indicating that a moderate occlusal load positively affects implant stability.

## Heat map analysis:

The heat map visualization (Figure 1 - see PDF) showed that:

[1] High-impact factors included gingival health and bone density, represented by darker green shading to indicate their strong influence on implant success.

[2] Moderate impact factors, such as occlusal load and prosthetic material, were represented in yellow.

[3] Low-impact factors, such as type of prosthesis, were represented in red, indicating less direct influence on implant stability.

## Implant success rate:

Overall, the study found that 90% of the implants had high stability and patient satisfaction scores. Patients with healthy gingiva, stable abutments, moderate occlusal load and medium to high bone density demonstrated the highest implant success rates, confirming the positive impact of combined prosthodontic and periodontal care on implant longevity.

## Discussion:

This study examines the role of prosthodontic and periodontal factors on implant success, particularly highlighting gingival health, occlusal load distribution, bone density and prosthetic material. The findings corroborate existing literature that emphasizes the importance of these factors for achieving optimal implant stability and longevity. Our findings underscore the importance of gingival health in implant success, where healthy gingiva significantly correlated with implant stability (r = 0.78, p < 0.01). Studies like those by Buser *et al.* (2013) [14] and other authors [15] have documented similar associations, reporting that maintaining healthy peri-implant tissue can reduce the risk of peri-implantitis, a common cause of implant failure. The study in literature [16, 17] also observed that patients with well-maintained gingival health had lower inflammation rates around implants, thereby enhancing long-term implant survival. The positive relationship between bone density and implant success (r = 0.72, p < 0.01) aligns with the classic findings in literature [18], who asserted that denser bone improves primary stability and facilitates better osseointegration. Our results echo with earlier reports [19], where denser bone was positively associated with implant stability, especially in the initial months post-placement. In a study in literature [20] the author added that higher bone density reduces micro-movement, supporting stable integration, which our study also observed in implants placed in areas with greater bone density. This study found that moderate occlusal load distribution enhanced implant stability, while excessive occlusal forces correlated with a lower success rate. A study [21, 22] reported similar findings, observing that well-balanced occlusal forces prevent excessive biomechanical stress on implants, which is critical for reducing marginal bone loss and ensuring implant longevity. Likewise, many authors [23] noted that excessive occlusal forces lead to adverse outcomes, such as implant micro-movement and component failure. This consistency with previous findings reinforces the importance of load management in implant design. While prosthetic material was moderately correlated with implant success in our study, ceramic and metal-ceramic materials showed better performance than zirconia. This finding is in agreement with a study [24], which highlighted that the choice of prosthetic material impacts stress distribution and biomechanical behavior around implants. In a review [25], metal-ceramic materials were shown to provide durability and stability, thereby contributing to improved implant longevity. This study further supports the evidence suggesting that selecting appropriate prosthetic materials can influence implant success and patient satisfaction. Additionally, longitudinal studies are recommended to assess how these factors influence implant stability over time, as suggested by authors [25], who noted that both biological and mechanical factors impacting implant stability can vary over time [26, 27].

## Conclusion:

The critical interplay between prosthodontic and periodontal factors in achieving dental implant success is shown. Healthy gingival tissues and adequate bone density emerged as strong predictors of implant stability, highlighting the importance of peri-implant care and precise pre-surgical planning. Additionally, moderate occlusal load distribution and the selection of durable prosthetic materials, such as ceramic or metal-ceramic, were shown to enhance implant longevity. These findings emphasize the need for an interdisciplinary approach that integrates prosthodontic functionality and periodontal health to optimize outcomes and improve patient satisfaction.

## Figures and Tables

**Table 1:** The key findings related to prosthodontic and periodontal factors in relation to implant success rates

**Factor**	**Categories/Values**	**Frequency (%)**	**Average Implant Stability**	**Correlation with Success**
Type of Prosthesis	Single Crown	50%	High	Moderate
	Bridge	30%	High	Moderate
	Overdenture	20%	Moderate	Low
Occlusal Load	Light	10%	Moderate	Low
	Moderate	60%	High	Moderate
	Heavy	30%	Moderate	Low
Prosthetic Material	Ceramic	40%	High	Moderate
	Metal-Ceramic	35%	High	Moderate
	Zirconia	25%	Moderate	Low
Abutment Stability	Stable	85%	High	High
	Unstable	15%	Low	Low
Gingival Health Status	Healthy	50%	High	High
	Mild Periodontitis	30%	Moderate	Moderate
	Severe Periodontitis	20%	Low	Low
Periodontal Pocket Depth	2.4 mm (Healthy)	-	High	High
	3.8 mm (Mild Periodontitis)	-	Moderate	Moderate
	5.6 mm (Severe Periodontitis)	-	Low	Low
Bone Density	Low	15%	Low	Moderate
	Medium	55%	High	High
	High	30%	High	High
